# A cognitively inspired feature-level fusion framework for interpretable retail sales forecasting using integration of extreme gradient boost machine, artificial neural network and attention mechanism model

**DOI:** 10.3389/fdata.2026.1889044

**Published:** 2026-08-20

**Authors:** Munienge Mbodila, Omobayo Ayokunle Esan

**Affiliations:** Department of Networking and Support, Walter Sisulu University, East London, South Africa

**Keywords:** big data, cognitively inspired feature-level fusion, interpretable retail sale forecasting, machine learning, retail sale forecasting

## Abstract

**Introduction:**

Accurate retail sales forecasting in modern data-intensive environments requires models that not only achieve high predictive accuracy but also scale efficiently as data volume and feature complexity increase. This study proposes a novel cognitively inspired hybrid framework, XGB–ANN–Attn, for interpretable and scalable retail analytics.

**Methods:**

The model introduces feature-level fusion by integrating XGBoost-derived leaf embeddings with deep neural representations, enabling joint modeling of low-order statistical dependencies and high-order nonlinear feature interactions. A lightweight attention mechanism dynamically assigns instance-specific feature importance, enhancing both predictive performance and interpretability while maintaining linear computational complexity with respect to feature dimensionality. Unlike conventional ensemble approaches that operate at the decision level, the proposed framework integrates representations at the level of representations, reducing redundancy and improving computational efficiency. The model is designed for scalability, combining the log-linear complexity of gradient boosting with the linear scaling properties of neural networks and attention mechanisms.

**Results:**

Experimental evaluation on the BigMart and Walmart datasets demonstrates superior performance compared to state-of-the-art models, achieving RMSE = 0.1584 and *R*^2^ = 0.9946 on BigMart, and RMSE = 0.8652 and *R*^2^ = 0.9568 on Walmart. Furthermore, the framework supports parallelization and distributed deployment, making it suitable for large-scale retail systems.

**Discussion:**

The alignment between attention weights and SHAP explanations provides transparent and actionable insights. The results confirm that the proposed approach offers a scalable, interpretable, and high-performance solution for AI-driven decision support systems in big-data retail environments.

## 1 Introduction

The rapid growth of data in modern retail environments has transformed decision-making processes, enabling organizations to leverage artificial intelligence (AI) for improved operational efficiency and strategic planning (Hsi-Peng Lu et al., 2023). Retail systems continuously generate large volumes of structured data from transactions, inventory records, and customer interactions, creating opportunities for data-driven optimization in areas such as demand forecasting, inventory management, and pricing strategies (Isharyani et al., 2024; Aktas and Meng, 2017). However, accurately modeling retail sales remains a challenging task due to nonlinear demand patterns, heterogeneous feature interactions, and dynamic operational conditions (Olatunji, 2025; Khedr and Rani, 2024).

Traditional statistical approaches and conventional machine learning models, including linear regression and tree-based methods, have been widely applied to retail forecasting (Mozher et al., 2025). While these models offer robustness and interpretability, they often struggle to capture complex, high-order relationships within structured data (Ajiga et al., 2024; Rezaei, 2025). Conversely, deep learning models provide powerful representation learning capabilities but may exhibit limitations when applied to tabular datasets, particularly in terms of interpretability, scalability, and sensitivity to data characteristics (Seyam et al., 2026; Sarker, 2021). As a result, recent research has explored hybrid approaches; however, most existing methods rely on decision-level ensembling, where model outputs are combined without fully exploiting complementary feature representations. In parallel, advances in tabular deep learning, such as attention-based architectures and transformer-inspired models, have introduced new mechanisms for modeling feature interactions (Jillani et al., 2025; Thivakaran and Ramesh, 2022). Despite their effectiveness, these approaches are often computationally expensive, require large-scale datasets, and may lack integration with tree-based models that are known to perform strongly on structured data (Martinović et al., 2025; Benard et al., 2025). Furthermore, many existing models provide limited interpretability, which restricts their practical adoption in real-world retail systems where transparency and trust are essential.

To address these limitations, this study proposes a novel hybrid predictive framework, termed XGB–ANN–Attn, that integrates tree-based ensemble learning and deep neural representation learning through feature-level fusion. Unlike conventional ensemble approaches, the proposed model combines XGB-derived leaf embeddings with neural feature representations at the representation level, enabling the joint modeling of low-order statistical dependencies and high-order nonlinear interactions. In addition, the framework incorporates a cognitively inspired attention mechanism, which dynamically assigns instance-specific importance weights to features. This mechanism is conceptually aligned with human selective attention, allowing the model to prioritize relevant information while suppressing less informative features. Beyond predictive performance, interpretability is a critical requirement for intelligent decision-support systems. The proposed framework integrates attention-based feature weighting with SHAP explainability, enabling transparent and consistent interpretation of model predictions. The alignment between attention weights and SHAP importance scores provides a novel perspective on explainability in hybrid models, bridging the gap between performance and interpretability. The proposed approach is designed with scalability in mind, leveraging the efficiency of gradient boosting and the flexibility of neural networks to support large-scale, data-driven retail applications. The model is evaluated on both the BigMart and Walmart datasets, representing structured and more complex retail scenarios, respectively. Experimental results demonstrate that the proposed framework consistently outperforms conventional machine learning models and state-of-the-art tabular learning approaches in terms of predictive accuracy, robustness, and generalization capability.

This study contributes to the advancement of AI-driven retail analytics by introducing a scalable, interpretable, and cognitively inspired through adaptive selective attention for structured data. By combining feature-level fusion, adaptive attention mechanisms, and explainability-driven design, the proposed model provides both methodological innovation and practical value for real-world retail decision-support systems. This study introduces a novel hybrid predictive framework that advances the state of the art in tabular retail analytics through feature-level model fusion and cognitively inspired attention mechanisms. The main contributions are as follows:

- Feature-level fusion for scalable hybrid learning: introduces a representation-level integration of XGBoost and neural networks, reducing redundancy and enabling efficient learning from large-scale structured data.
- Computationally efficient attention mechanism: develops a lightweight attention module with linear complexity, enabling adaptive feature weighting without the overhead of transformer-based architectures.
- Scalable architecture for big data environments: demonstrates that the proposed framework achieves near-linear scalability with respect to data size and feature dimensionality, supporting deployment in distributed systems.
- Explainability-performance alignment: integrates attention with SHAP to provide interpretable predictions without sacrificing accuracy.
- Robust cross-dataset validation: validates scalability and generalization across datasets with varying complexity, including structured and time-dependent retail data.

The proposed attention mechanism is conceptually grounded in principles of cognitive computing, particularly human selective attention, where individuals prioritize relevant information while filtering out less important stimuli. Similarly, the model dynamically adjusts feature importance based on context, enabling adaptive and interpretable decision-making. This cognitive-inspired design enhances the model's ability to focus on the most influential variables, thereby improving both predictive accuracy and transparency. Bridging machine learning with cognitive principles, the framework aligns with emerging trends in intelligent systems that emphasize human-like reasoning and explainability. The remainder of this paper is organized as follows. Section 2 presents the proposed methodology. Section 3 describes the experimental setup and evaluation metrics. Section 4 discusses the results and implications, and Section 5 concludes the study with future research directions.

## 2 Related works

The rapid growth of data in modern retail environments has enabled the adoption of artificial intelligence for improving operational efficiency and decision-making. In response, a substantial body of research has focused on AI-driven predictive analytics for retail sales forecasting, leveraging structured transactional datasets such as BigMart and Walmart. These datasets have been widely used to benchmark machine learning models for demand prediction and inventory optimization (Husban et al., 2025), (Jadhav and Jadhav, 2024). While these studies demonstrate the potential of data-driven approaches, they also reveal persistent challenges related to nonlinear demand patterns, heterogeneous feature interactions, and dynamic operational conditions, which remain insufficiently addressed in existing work. To tackle these challenges, early research primarily employed traditional statistical and machine learning models, including linear regression and basic supervised learning techniques. Studies such as Husban et al. (2025) and Jadhav and Jadhav (2024) report moderate forecasting accuracy; however, these approaches are inherently limited by their reliance on simplified assumptions and their inability to capture complex, higher-order feature interactions. As highlighted in subsequent analyses, such models often fail to generalize effectively across diverse retail scenarios, particularly when dealing with heterogeneous and high-dimensional data. In response to these limitations, researchers have increasingly adopted ensemble learning methods, particularly tree-based approaches such as random forest and XGB. Works such as Manasa et al. (2026) and Ismail et al. (2025) demonstrate that these models significantly improve predictive performance by capturing nonlinear relationships and reducing variance. Despite their effectiveness, these methods typically operate at the decision level, combining outputs from multiple models without integrating their internal feature representations. As a result, they do not fully exploit complementary information across different learning paradigms, limiting their ability to model complex interactions in a unified manner.

Parallel to advances in machine learning, deep learning approaches have been introduced to enhance representation learning capabilities. Neural network-based models, including hybrid architectures for retail forecasting (Hossam et al., 2023), (Mansur et al., 2025), have shown strong potential in capturing high-order nonlinear dependencies. However, when applied to structured tabular data, these models exhibit notable limitations, including sensitivity to data characteristics, high computational requirements, and limited interpretability. Furthermore, their performance often depends on large-scale datasets, making them less effective in moderate-sized retail datasets such as BigMart. More recently, advances in tabular deep learning, particularly attention-based and transformer-inspired architectures (Hollmann et al., 2025), have introduced new mechanisms for modeling feature interactions. These models leverage attention to capture dependencies across features, offering improved flexibility compared to traditional approaches. Nevertheless, they are often computationally expensive, require extensive hyperparameter tuning, and lack integration with tree-based models, which remain highly effective for structured data. This separation between tree-based and neural approaches highlights a critical limitation in current research. To bridge the gap between machine learning and deep learning, several studies have explored hybrid modeling strategies. While these approaches aim to combine the strengths of different models, most existing methods rely on decision-level ensembling techniques, such as averaging or stacking predictions. This design limits their ability to learn joint feature representations, thereby underutilizing the complementary strengths of ensemble learning and neural networks. Additionally, existing hybrid approaches rarely incorporate mechanisms for adaptive, instance-specific feature weighting, which is essential for modeling dynamic retail environments where feature importance varies across contexts. Another critical limitation across existing studies is the lack of interpretability. Although some research has focused on explainable machine learning (Bajaj et al., 2020), these approaches often involve trade-offs between transparency and predictive performance. In real-world retail applications, where decisions impact inventory management, pricing, and supply chain operations, models must provide both high accuracy and interpretable insights. Current approaches do not adequately address this dual requirement. Table 1 provides a summary of recent related works on retail sales forecasting

**Table 1:** Summary of recent related works.

Study	Problem addressed	Method used	Dataset used	Limitations
Husban et al. (2025)	Sales forecasting accuracy	Regression machine learning	BigMart	Poor missing data handling
Manasa et al. (2026)	Demand prediction	Random forest (RF), XGB	BigMart	No seasonality modeling
Jadhav and Jadhav (2024)	Model comparison	Multiple machine learning models	BigMart (Kaggle)	No real-time data
Ismail et al. (2025)	Forecast accuracy	Ensemble machine learning	BigMart	Scalability issues
Wen et al. (2024)	Retail optimization	XGBoost, *k*-nearest neighbor (*k*-NN)	BigMart	Low interpretability
Singh (2023)	Sales pattern prediction	Principal component analysis (PCA) + *k*-nearest neighbor (*k*-NN) + decision tree (DT)	BigMart	Overfitting risk
Jillani et al. (2025)	Price & sales prediction	machine learning models	BigMart	No external factors
Padma et al. (2023)	Inventory optimization	XGB, support vector regression (SVR)	BigMart	No deep learning
Hobor et al. (2025)	Retail demand irregularities	Ensemble + artificial neural network (ANN)	Retail datasets	Neural inefficiency
Ganguly and Mukherjee (2024)	Forecast optimization	Random forest	Retail data	High computation
Haque et al. (2023)	Demand forecasting	ML + macroeconomic data	Retail data	Complex data requirements
Mansur et al. (2025)	Forecast accuracy	Deep learning	Retail time-series	Needs large data
Bajaj et al. (2020)	Explainable prediction	Interpretable ML	Retail data	Lower accuracy
Hollmann et al. (2025)	Tabular prediction	Transformer-based model	Tabular datasets	Limited scalability

To address these limitations, this study proposes a cognitively inspired hybrid framework (XGB–ANN–Attn) that aligns directly with the identified challenges. The model introduces a feature-level fusion mechanism that integrates XGB-derived leaf embeddings with neural representations, enabling the joint modeling of low-order statistical dependencies and high-order nonlinear interactions, thereby addressing the limitations of both traditional machine learning and deep learning approaches. The framework incorporates a cognitively inspired attention mechanism that dynamically assigns instance-specific importance to features. This directly addresses the need for adaptive modeling of heterogeneous feature interactions, as highlighted in prior work. Finally, the proposed approach integrates attention-based learning with SHAP explainability, ensuring alignment between predictive performance and interpretability. This resolves the longstanding trade-off between accuracy and transparency, providing actionable insights for real-world retail decision-support systems.

### 2.1 Distinction from existing deep tabular models

Recent advances in tabular deep learning have produced several high-performing architectures, including TabNet, NODE, FT-Transformer, SAINT, DeepGBM, and TabTransformer. These models have demonstrated strong capability in modeling nonlinear feature interactions within structured datasets. However, important limitations remain with respect to scalability, interpretability, computational efficiency, and integration between heterogeneous learning paradigms. TabNet employs sequential attention for feature selection and representation learning; however, its performance is highly dependent on extensive hyperparameter optimization and large-scale training data. Furthermore, TabNet relies exclusively on neural representation learning and does not explicitly incorporate tree-based structural information. Transformer-based approaches such as FT-Transformer and TabTransformer model feature relationships using self-attention mechanisms. While effective, these approaches typically exhibit high computational complexity and may require substantial computational resources for training and deployment. Their scalability becomes increasingly challenging in high-dimensional retail datasets involving millions of records. DeepGBM and related hybrid architectures attempt to integrate gradient boosting with neural networks. Nevertheless, most existing approaches rely on either decision-level fusion or loosely coupled architectures that do not fully exploit complementary feature representations.

The proposed XGB–ANN–Attn framework differs from existing approaches in several important aspects:

- The framework performs feature-level fusion rather than decision-level ensembling, enabling interaction between tree-based and neural representations within a unified latent feature space.
- The proposed attention mechanism operates with linear computational complexity, reducing the overhead commonly associated with transformer-based self-attention.
- The framework explicitly aligns attention-based feature weighting with SHAP explainability, improving interpretability and transparency.
- The architecture is designed for scalable deployment in distributed retail analytics environments.
- The proposed framework integrates complementary inductive biases from ensemble learning and neural representation learning while maintaining computational efficiency.

Unlike purely neural tabular architectures, the proposed approach leverages the strong structured-data learning capabilities of gradient boosting while simultaneously benefiting from deep nonlinear representation learning. This combination enables improved generalization performance across heterogeneous retail datasets.

### 2.2 Theoretical motivation for feature-level fusion

**Figure 1:**
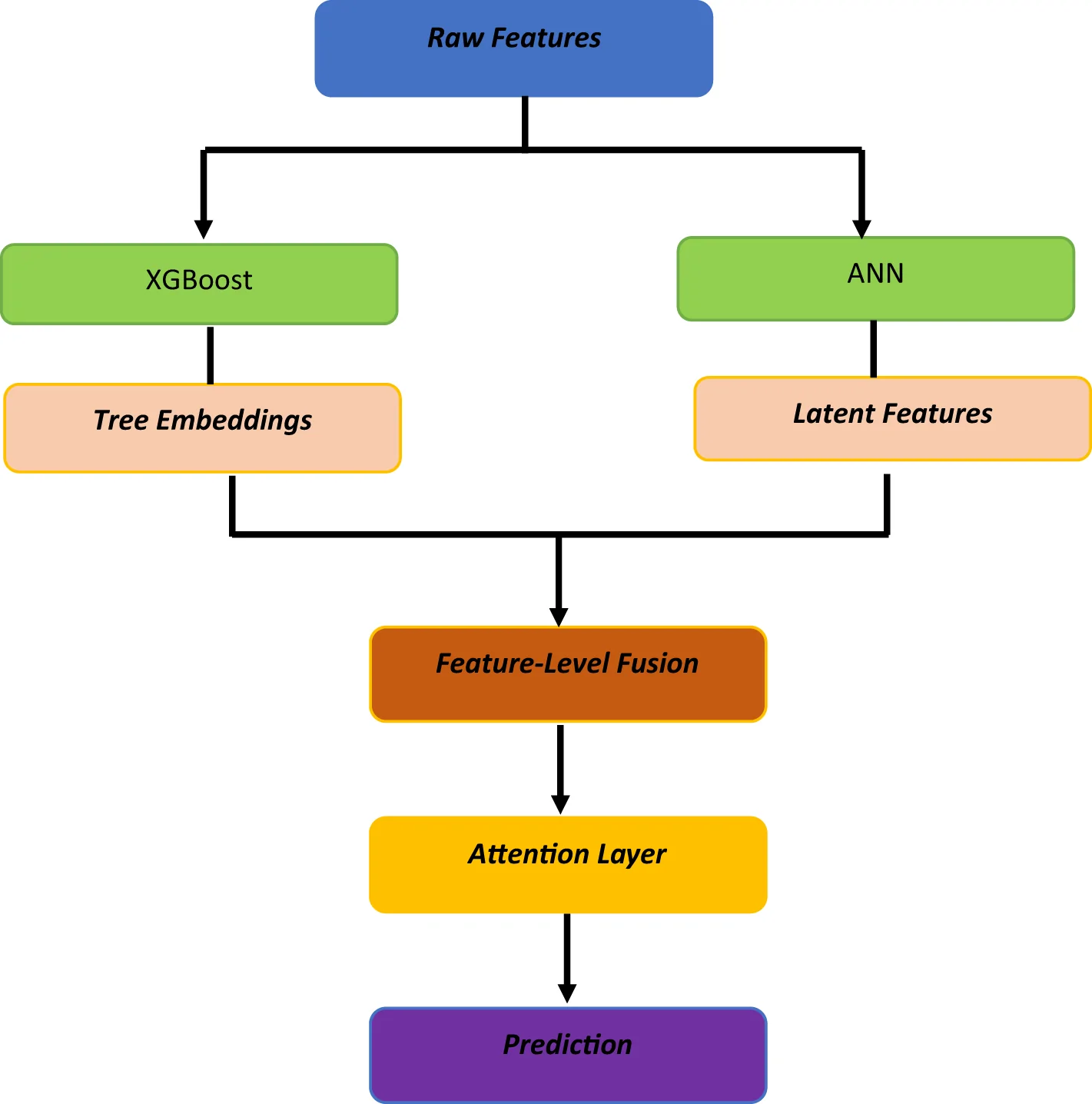
Proposed model feature-fusion level.

The motivation for combining XGBoost and artificial neural networks arises from their complementary inductive biases. XGBoost constructs an ensemble of decision trees that partitions the feature space into locally homogeneous regions, making it highly effective for structured tabular data, handling missing values, nonlinear relationships, and feature interactions. However, tree-based methods learn piecewise decision boundaries that may not adequately model smooth, high-order nonlinear relationships. Artificial neural networks, in contrast, learn continuous hierarchical feature representations through nonlinear activation functions. Their distributed representations allow the discovery of latent interactions that are difficult to capture using tree-based partitions alone. Nevertheless, ANNs often require extensive training data and may underperform on structured tabular datasets. The proposed framework exploits the complementary strengths of both learning paradigms by integrating XGBoost leaf embeddings with neural representations before prediction. Rather than combining predictions after learning (decision-level fusion), the proposed method performs representation-level fusion, enabling interaction between tree-derived structural information and learned latent features. This richer representation increases the information available for prediction while reducing redundancy, thereby improving generalization performance. An illustration of the proposed model feature-level fusion is shown in Figure 1.

## 3 Method

**Figure 2:**
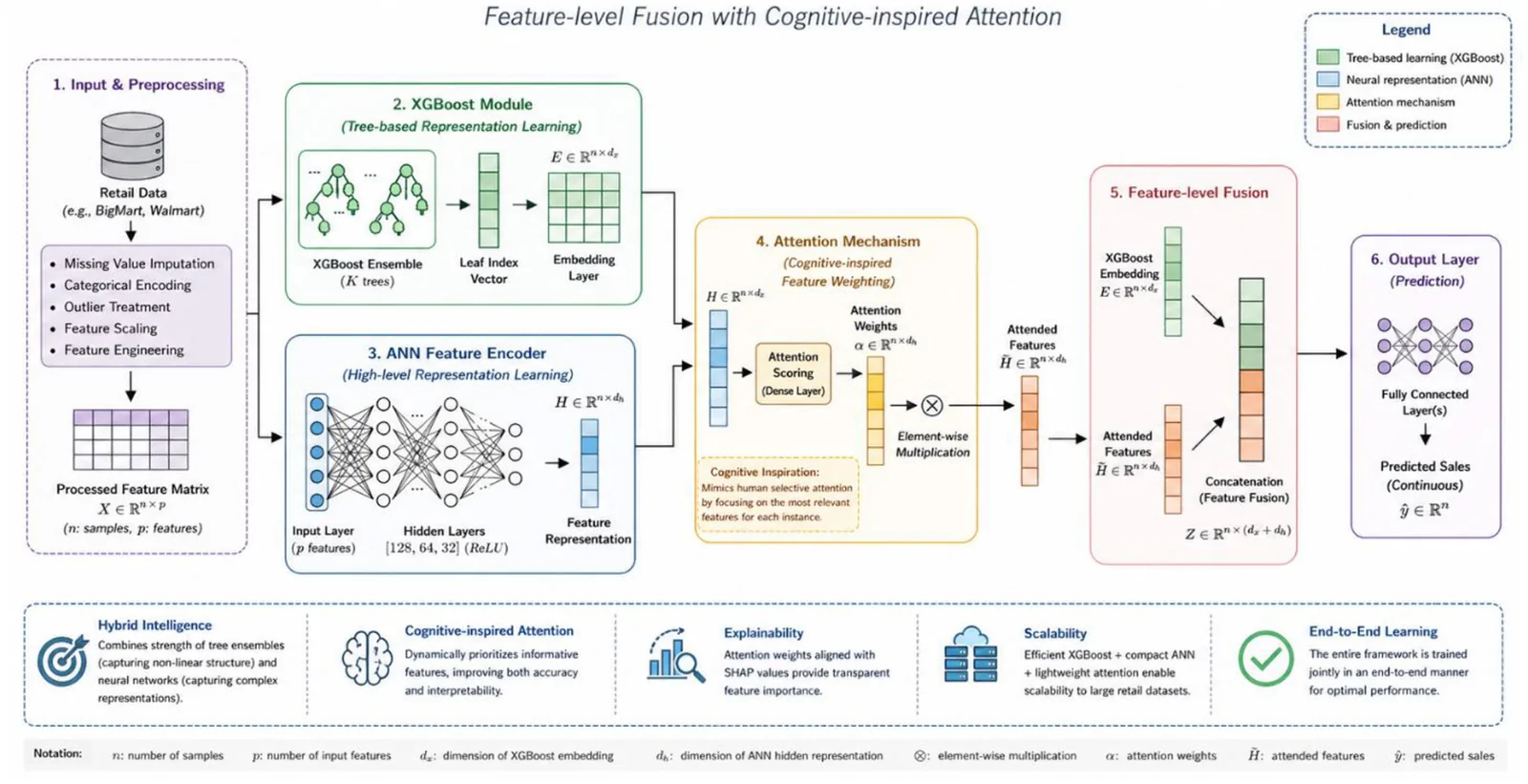
AI-driven retail sales forecasting system model.

The framework is designed following best practices in machine learning pipelines, integrating data acquisition, preprocessing, feature engineering, and model development. The proposed analytical framework is shown in Figure 2.

Figure 2 illustrates the scalable architecture of the proposed framework, highlighting its integration within a distributed big data ecosystem. The design supports parallel processing, real-time analytics, and efficient handling of high-volume retail data.

### 3.1 Data acquisition

Data acquisition represents a foundational component of the analytical pipeline, directly influencing the reliability, validity, and generalizability of subsequent modeling outcomes. This study utilizes a secondary dataset sourced from the publicly available BigMart sales repository hosted on the Kaggle platform https://www.kaggle.com/datasets/shivan118/big-mart-sales-prediction-datasets. Also, the Walmart sales forecast dataset was used for generalization of the model, and it can be found in https://www.kaggle.com/datasets/aslanahmedov/walmart-sales-forecast. We also used a big dataset of M5 sales Walmart retail good dataset found in https://www.kaggle.com/competitions/m5-forecasting-accuracy/data. These datasets provide a structured representation of retail transactions, enabling reproducible and transparent empirical analysis. The dataset was obtained from the Kaggle data science platform, which hosts curated datasets for research and benchmarking purposes. The BigMart dataset has been widely adopted in predictive analytics studies, making it suitable for comparative evaluation of machine learning models. The dataset is provided in comma-separated values (CSV) format, facilitating interoperability across analytical environments. Its public availability ensures reproducibility of results, transparency in methodological implementation, and benchmarking against existing studies. No proprietary or confidential data was used, eliminating restrictions related to data access or sharing.

The selected datasets were chosen because they capture diverse aspects of consumer purchasing behavior. The BigMart dataset represents customer purchasing decisions across multiple product categories and outlet characteristics. The Walmart dataset introduces temporal variations, promotional activities, and seasonal purchasing behavior, while the M5 dataset provides large-scale transactional records collected from multiple retail stores over extended periods. Collectively, these datasets represent heterogeneous consumer demand patterns and therefore provide a realistic benchmark for evaluating retail sales forecasting models. The implemented datasets with the consumer characteristics are shown in Table 2.

**Table 2:** Datasets with consumer characteristics.

Dataset	Consumer characteristics
BigMart	Product preference, outlet choice, pricing behavior
Walmart	Weekly demand, promotions, seasonal buying
M5	Large-scale customer purchasing trends

#### 3.1.1 Feature description

The dataset includes the following key variables: product-Level Attributes (such as Item_Weight: continuous variable representing product weight, Item_Fat_Content: categorical variable indicating fat content classification, Item_Visibility: continuous variable representing product display visibility, Item_Type: categorical variable describing product category, Item_MRP: continuous variable representing maximum retail price), Outlet-Level Attributes (such as Outlet_Identifier: unique identifier for each retail outlet, Outlet_Establishment_Year: year of outlet establishment, Outlet_Size: categorical variable indicating outlet size, Outlet_Location_Type: categorical variable describing location tier, Outlet_Type: categorical variable specifying outlet format) and Target Variable (such as Item_Outlet_Sales: continuous variable representing sales revenue for each product-outlet pair). The selected dataset attributes used for the implementation in this research are shown in Table 3.

**Table 3:** Attributes and variables of the dataset.

Item identifier	ProductID
Item weight	Continuous variable representing product weight
Item_Fat_Content	Categorical variable indicating fat content classification
Item_Visibility	Continuous variable representing product display visibility
Item_Type	Categorical variable describing product category
Item_MRP	Continuous variable representing maximum retail price
Outlet_Identifier	Unique identifier for each retail outlet
Outlet_Establishment_Year	Year of outlet establishment
Outlet_Size	Categorical variable indicating outlet size
Outlet_Location_Type	Categorical variable describing location tier
Outlet_Type	Categorical variable specifying outlet format
Item_Outlet_Sales	Continuous variable representing sales revenue for each product-outlet pair

### 3.2 Data preprocessing

Data preprocessing constitutes a critical stage in the machine learning pipeline, ensuring data quality, consistency, and suitability for predictive modeling. Given the heterogeneous structure of the BigMart dataset, a systematic preprocessing framework was implemented, comprising missing value imputation, categorical standardization, feature engineering, encoding, scaling, and outlier treatment.

#### 3.2.1 Missing value imputation

The dataset exhibits non-trivial missingness in key variables, specifically ItemWeight (17.16%) and Outlet_Size (28.28%). Appropriate imputation strategies were employed to minimize bias while preserving the underlying data distribution. For numerical variables with skewed distributions, median imputation is preferred due to its robustness to outliers. To minimize bias introduced by missing numerical values while preserving the original distribution of each product category, group-wise median imputation was adopted. Unlike mean imputation, the median is less sensitive to extreme values and therefore provides a more robust estimate for skewed retail data, as in Equation 1.


$${\tilde{x}}_{i}=\{\begin{array}{ll} x_{i}, & if\;x_{i}is\;observed\\ median\left(X_{j}\right),if\;x_{i}\;is\;missuing,\;where\; & j\in Item\_Type \end{array}$$ (1)

Where ItemWeight_i_ denotes the missing value for the i^th^ observation, and Median (ItemType) represents the median weight computed within the corresponding product category. This approach preserves intra-category characteristics and reduces the distortion that may arise from global imputation.

This group-wise imputation preserves intra-category variability and reduces distortion compared to global imputation. Given the higher proportion of missingness and the categorical nature of Outlet_Size, predictive imputation was applied using a supervised learning approach. Missing values in the Outlet_Size variable cannot be reliably estimated using simple statistical measures because the variable is categorical. Therefore, a supervised classification model is employed to infer the most probable outlet size from correlated outlet characteristics. A classification model *f*(*X*) was trained on observed instances using Equation 2.


$$\begin{array}{l} ŷ=f\left(X\right)=\underset{c\varepsilon C}{\text{argmax}}P\left(y=c\;|X\right) \end{array}$$ (2)

Where *c* represents possible outlet size categories. Predictor variables included Outlet_Type, Outlet_Location_Type, and Outlet_Establishment_Year. This approach leverages structural relationships in the data to infer missing categories. This formulation exploits relationships between outlet type, location, and establishment year to estimate missing outlet-size categories. Consequently, the imputed values remain consistent with the structural characteristics of the retail environment.

Inconsistencies in categorical labels can introduce redundancy and reduce model performance. For instance, the Item_Fat_Content variable contained multiple representations of equivalent categories (such as “LF,” “Low,” “Fat,” “low fat”). A map function *g*(*x*) was applied to standardize categories as in Equation 3.


$$\begin{array}{l} x^{\prime }=g\left(x\right) \end{array}$$ (3)

Where *g*(*x*)∈{*Low Fat, Regular*}. This transformation ensures semantic consistency and reduces dimensionality during encoding.

### 3.3 Exploratory data analysis (EDA)

Exploratory data analysis plays a crucial role in identifying key characteristics, examining distributions, and exploring relationships between variables. This process enables us to explore the key qualities and patterns present in the dataset in greater detail. Exploratory data analysis becomes especially significant when dealing with large datasets, as it uncovers the hidden patterns and traits that often lie just beneath the surface of the data (Dhummad, 2025). To make the data easier to interpret, visualizations such as stacked bar charts, vertical bar graphs, and horizontal bar graphs were employed to gather patterns, trends, and anomalies in the dataset. Statistical analysis was performed on the dataset to help understand the statistical distribution of both categorical and numerical features. Additionally, a heatmap was plotted to show the correlation between the numerical features.

#### 3.3.1 Descriptive statistical analysis

The dataset consists of *N* = 8,523N observations with a mixture of numerical and categorical variables. For each numerical variable *X*, standard descriptive statistics are computed, including mean, variance, standard deviation, skewness, and kurtosis. The sample mean $\left(\bar{X}\right)$ is defined as in Equation 4.


$$\begin{array}{l} \bar{X}=\frac{1}{N}\overset{N}{\underset{i=1}{\sum }}X_{i} \end{array}$$ (4)

#### 3.3.2 Correlation analysis

Correlation analysis is performed to quantify the strength and direction of linear relationships between numerical variables before model development. To quantify relationships between numerical variables, the Pearson correlation coefficient is computed in Equation 5.


$$\begin{array}{l} r_{XY}=\frac{\sum_{i=1}^{N}\left(X_{i}-\bar{X}\right)\left(Y_{i}-\bar{Y}\right)}{\sqrt{\sum_{i=1}^{N}{\left(X_{i}-\bar{X}\right)}^{2}\sum_{i=1}^{N}{\left(Y_{i}-\bar{Y}\right)}^{2}}} \end{array}$$ (5)

**Figure 3:**
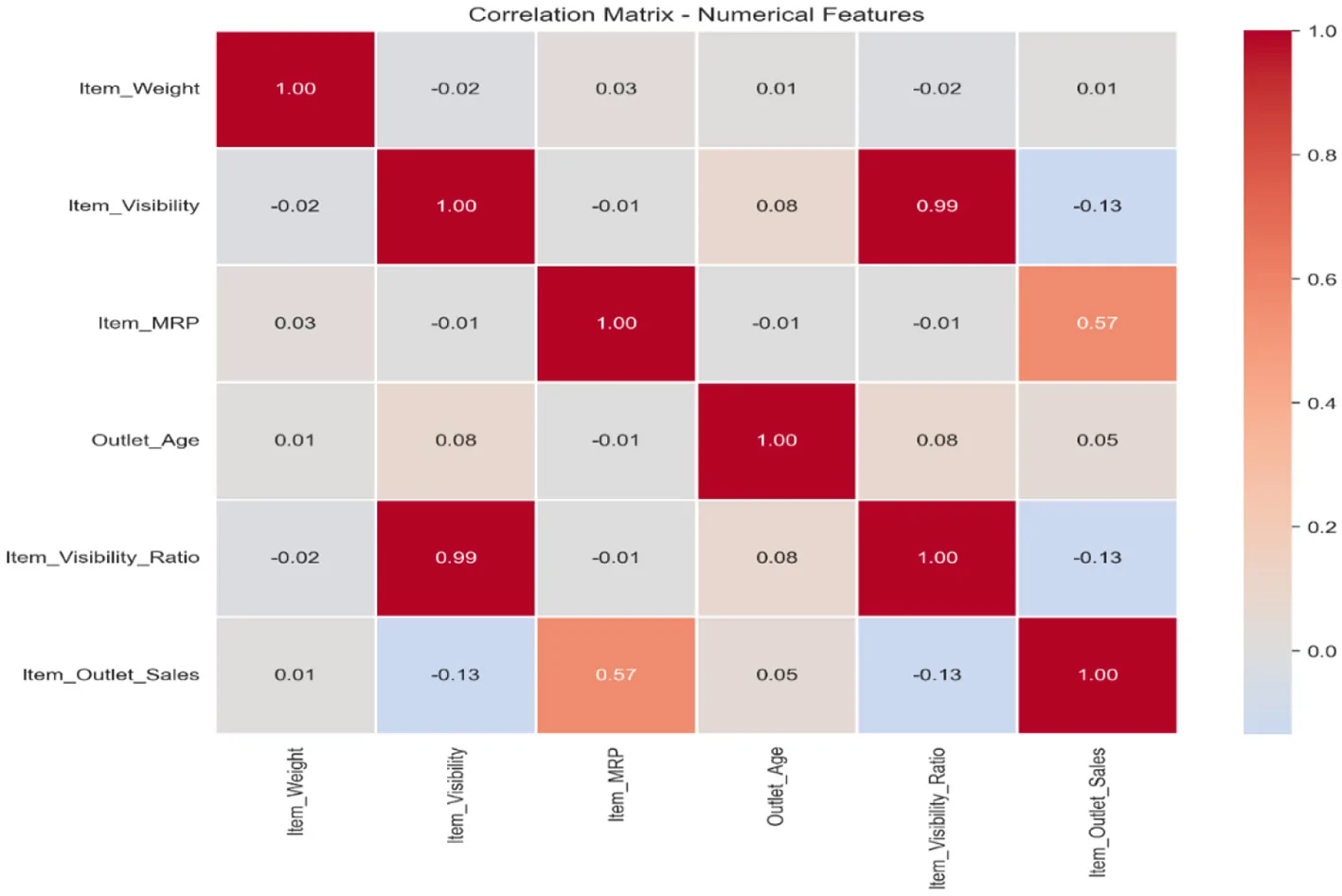
Correlation analysis captures complex interactions within the dataset.

The Pearson correlation coefficient ranges from −1 to +1, where positive values indicate direct relationships and negative values indicate inverse relationships. Variables exhibiting weak linear relationships may still contribute to prediction because nonlinear interactions are subsequently captured by XGBoost and the neural network. The correlation matrix of Item Weight, Item Visibility, Item_MRP, and Item_Outlet_Sales is displayed as a heatmap in Figure 3.

There is a somewhat favorable association (0.57) between Item_MRP and Item_Outlet_Sales. There is little link between Item Weight and Item Visibility and other attributes. The strength of the correlation is indicated by the color intensity. Correlation analysis reveals a strong positive correlation between Item_MRP and Item_Outlet_Sales, weak correlations between Item_Visibility and Sales, and minimal linear relationships among several predictors. These findings suggest that nonlinear models may be appropriate for capturing complex interactions within the dataset.

### 3.4 Feature engineering

Feature engineering enhances model performance by extracting informative attributes from raw data. The attributes include Outlet_Age, Price Binning, and Visibility Adjustment. The operational age of each outlet was derived as in Equation 6.


$$\begin{array}{l} Outlelet\text{\_}Age=Y_{current}-Y_{establishment} \end{array}$$ (6)

Where *Y*_*current*_ is the reference year of the analysts, this feature captures lifecycle effects on sales performance. The continuous Item_MRP variable was discretized into bins to capture nonlinear price effects as in Equation 7.


$$\begin{array}{l} B_{k}=\left\{x\;|\;t_{k-1}\leq x\leq t_{k}\right\} \end{array}$$ (7)

Where *t*_*k*_ are the bin thresholds. This transformation enables models to capture segmented pricing behavior. Zero values in Item_Visibility may represent missing or anomalous entries. These were replaced with the mean visibility, which is computed in Equation 8.


$$x_{i}=\{\begin{array}{c} x_{i},\;if\;x_{i}>0\\ \mu_{j},\;if\;x_{i}=0 \end{array}$$ (8)

Where μ_*j*_ is the mean visibility for category *j*.

#### 3.4.1 Encoding of categorical variables

Machine learning algorithms require numerical inputs; therefore, categorical variables were encoded appropriately. The nominal variables were transformed using one-hot encoding using Equation 9.


$$x_{ij}=\{\begin{array}{cc} 1, & if\;observation\;i\;belongs\;to\;j\\ 0, & otherwise \end{array}$$ (9)

This results in a binary matrix representation, preserving category independence.

#### 3.4.2 Feature scaling

Feature scaling was applied to ensure that variables contribute equally to model training, particularly for distance-based and gradient-based algorithms. Furthermore, *z*-score normalization is used for feature scaling, as computed in Equation 10. This transformation produces features with zero mean and unit variance.


$$\begin{array}{l} z=\frac{x-\mu }{\sigma } \end{array}$$ (10)

Where μ is the mean and σ is the standard deviation. The outliers were analyzed using statistical thresholds based on the interquartile range (IQR) in Equations 11, 12.


$$\begin{array}{l} IQR=Q_{3}-Q_{1} \end{array}$$ (11)

$$\begin{array}{l} Outlier\;if\;x\;<Q_{1}-1.5\;\cdot IQR\;or\;x\;>Q_{3}+1.5\;\cdot IQR. \end{array}$$ (12)

However, unlike typical data cleaning pipelines, legitimate retail outliers (such as high sales volume during peak demand) were retained to preserve real-world variability and avoid biasing model predictions. The dataset was partitioned into training and testing subsets using Equation 13.


$$\begin{array}{l} D=D_{train}\cup D_{test},D_{train}\cap D_{test}=\emptyset \end{array}$$ (13)

Typically, an 80:20 was used. Additionally, *k*-fold cross-validation was applied, as in Equation 14.


$$\begin{array}{l} CV_{k}=\frac{1}{k}\overset{k}{\underset{i=1}{\sum }}L\left(f_{-i},D_{i}\right) \end{array}$$ (14)

Where L denotes the loss function and *f*_−*i*_ is the model trained excluding fold *i*.

### 3.5 Model development: XGB-ANN-Attn framework

To model complex nonlinear relationships in retail sales data while ensuring scalability and interpretability, this study proposes a hybrid model that integrates tree-based learning, neural representation learning, and attention-based feature weighting at the representation level. Let the input dataset be defined as in Equation 15.


$$\begin{array}{l} D={\left\{\left(x_{i},y_{i}\right)\right\}}_{i=1}^{n},\;x_{i}\in {\mathbb{R}}^{d},\;y_{i}\in \mathbb{R} \end{array}$$ (15)

Where *x*_*i*_ represents the feature vector and *y*_*i*_ target sales value.

#### 3.5.1 XGB representation learning

XGBoost serves as the first representation-learning component of the proposed hybrid framework. Rather than generating predictions alone, the model learns tree-based structural representations that capture nonlinear interactions and partition the feature space into locally homogeneous regions. An extreme gradient boosting model is trained to learn nonlinear feature interactions using Equation 16.


$$\begin{array}{l} f_{XGB}\left(x\right)=\overset{T}{\underset{t=1}{\sum }}f_{k}\left(x\right),\;f_{k}\;\in \;F \end{array}$$ (16)

where T denotes the number of boosting trees and *f*_*k*_ represents the prediction function of the k^th^ tree. Each successive tree attempts to minimize the residual errors produced by the previous ensemble. Consequently, XGBoost learns robust local decision structures that are later fused with neural representations.

Each sample follows a unique path through the ensemble of decision trees and arrives at a set of terminal leaf nodes. These leaf indices are converted into dense embedding vectors so that structural information learned by XGBoost can be integrated with neural representations. This embedding process transforms discrete tree outputs into continuous representations suitable for deep learning using Equation 17.


$$\begin{array}{l} z_{i}^{\left(xgb\right)}=embed\left(LeafIndices\left(x_{i}\right)\right)\in^{d_{1}} \end{array}$$ (17)

This embedding captures low-order statistical dependencies and tree-based feature interactions.

#### 3.5.2 Neural representation learning

The neural network is responsible for learning continuous latent feature representations that complement the partition-based representations learned by XGBoost. Multiple nonlinear transformations enable the extraction of complex feature interactions that may not be captured by decision trees alone. The sample input *x*_*i*_ is passed through a feedforward neural network, as in Equations 18–20.


$$\begin{array}{l} h^{\left(1\right)}=\sigma \left(W_{1}x_{i}+b_{1}\right) \end{array}$$ (18)

$$\begin{array}{l} h^{\left(l\right)}=\sigma \left(W_{1}h^{\left(l-1\right)}+b_{l}\right),\;l=2,\ldots .,L \end{array}$$ (19)

$$\begin{array}{l} z_{i}^{\left(ann\right)}=h^{\left(L\right)}\in {\mathbb{R}}^{d_{2}} \end{array}$$ (20)

Where σ is a nonlinear activation (ReLU) and *W*_*l*_*b*_*l*_ are learnable parameters. This captures high-order nonlinear feature interactions. ReLU activation introduces nonlinearity while preventing vanishing gradients during optimization. The resulting representation captures higher-order interactions among retail attributes and provides complementary information for the subsequent feature-fusion stage

#### 3.5.3 Attention mechanism

To dynamically assign feature importance, an attention function is defined over the neural representation as follows:

Before Equation 21

Inspired by the theory of human selective attention, the proposed attention mechanism dynamically estimates the relative importance of each learned feature representation. Instead of treating every feature equally, the model allocates greater emphasis to informative features while suppressing irrelevant information.

After Equation 21

Equation 21 computes the raw attention score for every feature representation.

**Steps 1**: attention score computation as in Equation 21.


$$\begin{array}{l} e_{i}=W_{a}z_{i}^{\left(ann\right)}+b_{a} \end{array}$$ (21)

Equation 21 computes the raw attention scores for each learned feature representation. Rather than assigning equal importance to all features, the attention mechanism estimates their relative contribution to the prediction task. Features receiving higher attention scores are considered more informative for forecasting retail sales.

**Step 2:** normalization (Softmax) is computed as in Equation 22.


$$\begin{array}{l} \alpha_{ij}=\frac{exp\left(e_{ij}\right)}{\sum_{k=1}^{d_{2}}exp\left(e_{ik}\right)} \end{array}$$ (22)

Where α_*ij*_ represents the importance of feature *j* for instance *i*, $\sum_{j}\alpha_{ij}=1$. Equation 22 normalizes the attention scores using the Softmax function so that the weights form a probability distribution whose values sum to one. This enables the network to interpret the attention values as relative feature importance.

**Step 3:** attention-weighted representation is computed using Equation 23.


$$\begin{array}{l} {\bar{z}}_{i}^{\left(ann\right)}=\alpha_{i}\;⊙\;z_{i}^{\left(ann\right)} \end{array}$$ (23)

Where ⊙ denotes element-wise multiplication. This formulation ensures instance-specific, context-aware feature weighting with linear complexity 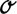
(*d*). The weighted representation is obtained by multiplying each feature vector by its corresponding attention weight. Features assigned larger weights contribute more strongly to the final prediction, thereby improving both interpretability and prediction accuracy.

#### 3.5.4 Feature-level fusion

Feature-level fusion constitutes the central innovation of the proposed framework. Unlike decision-level ensemble methods that combine only final predictions, the proposed approach integrates intermediate feature representations generated by XGBoost and the neural network, as in Equations 24, 25.


$$\begin{array}{l} z_{i}=\phi \left(\left[z_{i}^{\left(xgb\right)}||{\bar{z}}_{i}^{\left(ann\right)}\right]\right) \end{array}$$ (24)

Where || denotes concatenation and ϕ(.) is a projection function (fully connected layer).


$$\begin{array}{l} z_{i}=\sigma \left(W_{f}\left[z_{1}^{\left(xgb\right)}||\;{\tilde{z}}_{i}^{\left(ann\right)}\right]\;+b_{f}\right) \end{array}$$ (25)

Concatenation preserves complementary information extracted by both learning paradigms, while the projection layer learns joint latent representations. This interaction enables the discovery of relationships that neither individual model can learn independently.

#### 3.5.5 Prediction layer

The final prediction is computed as in Equation 26.


$$\begin{array}{l} {ŷ}_{i}=W_{0}z_{i}+b_{0} \end{array}$$ (26)

The prediction layer transforms the fused representation into the final retail sales estimate. The predicted value therefore reflects both tree-based structural information and deep nonlinear representations simultaneously.

#### 3.5.6 Loss function and optimization

Model optimization aims to minimize prediction errors by adjusting the learnable neural network and attention parameters. The model is trained using mean square error in Equation 27.


$$\begin{array}{l} L=\frac{1}{n}\overset{n}{\underset{i=1}{\sum }}{\left(y_{i}-{ŷ}_{i}\right)}^{2} \end{array}$$ (27)

Mean Squared Error penalizes large prediction errors more heavily than small errors, making it particularly suitable for retail forecasting where substantial forecasting mistakes can significantly affect inventory and supply-chain decisions. The step-by-step procedure for the proposed method is illustrated in Algorithm 1.

**Algorithm 1:** XGB-ANN-Attn training.

**Input:** Dataset *D* = {(*x*_*i*_, *y*_*i*_)}
1. Train XGBoost model *onD*
2. Extract leaf indices → computer *z*^(*xgb*)^
3. Feed pass x through ANN → computer *z*^(*ann*)^
4. Compute attention-weighted α using softmax
5. Computer attention-weighted features: *z*^(*ann*)^
6. Concatenate: *z* = [*z*^(*xgb* )^||*z*^(*ann*)^]
7. Apply fusion layer
8. Predict output ŷ
9. Compute loss (MSE)
10. Update ANN + attention parameters via backpropagation

#### 3.5.7 Hyperparameter tuning

The performance of machine learning and hybrid models is highly sensitive to the choice of hyperparameters, particularly in architectures that combine heterogeneous components such as gradient boosting, neural networks, and attention mechanisms. To ensure a fair and robust evaluation, a systematic hyperparameter optimization process was conducted for all models considered in this study. For the proposed XGB–ANN–Attn framework, key parameters, including the number of boosting trees, tree depth, learning rate, neural network architecture (number of layers and hidden units), and attention dimensionality, were carefully tuned using cross-validation. This process aims to balance model complexity and generalization performance while avoiding overfitting. Baseline models were also tuned using comparable search strategies to ensure consistency and fairness in comparison. The final set of selected hyperparameters reflects configurations that achieved optimal performance on validation data while maintaining computational efficiency. The optimal hyperparameter settings used in the experiments are summarized in Table 4.

**Table 4:** Hyperparameter tuning.

Component	Parameter	Value
XGB	n_estimators	300
XGB	max_depth	6
XGB	learning_rate	0.05
XGB	subsample	0.8
ANN	Hidden layers	(128; 64; 32)
ANN	Activation	ReLU
ANN	Dropout	0.3
Training	Optimizer	Adam
Training	Learning rate	0.001
Training	Batch size	32
Training	Epochs	100
Attention	Type	Dense + Softmax
Validation	*K*-fold	5

#### 3.5.8 Theoretical justification of feature-level fusion

The proposed feature-level fusion strategy can further be interpreted through an information-theoretic perspective. Let *X* denote the input feature space and *Y* denote the target prediction variable. The objective of representation learning is to maximize the mutual information between the learned representation *Z* and the target variable *Y*. Tree-based models and neural networks learn different functional mappings of the input space. Gradient boosting primarily captures partition-based local decision boundaries, while neural networks learn smooth nonlinear transformations through hierarchical feature abstraction. Consequently, the representations generated by these models contain partially complementary information. Let *Z*_*xgb*_ represent the tree-based embedding space and *Z*_*ann*_ represent the neural representation space. The fused representation is defined as in Equation 28.


$$\begin{array}{l} Z_{fusion}\;=\;f\left(Z_{xgb}\;||\;Z_{ann}\right) \end{array}$$ (28)

where || denotes concatenation and *f*(.) represents a nonlinear projection function.

The fused representation increases the effective mutual information with the target variable according to Equation 29 under the assumption that the individual representations contain non-redundant information.


$$\begin{array}{l} I\left(Z_{fusion}\;;\;Y\right)\;\geq \;\max\left(I\left(Z_{xgb}\;;\;Y\right),\;I\left(Z_{ann}\;;\;Y\right)\right) \end{array}$$ (29)

This theoretical interpretation explains why feature-level fusion improves predictive performance relative to standalone models. Integrating heterogeneous representations, the framework increases representational richness while preserving complementary structural information. Furthermore, the attention mechanism can be interpreted as a data-dependent regularization operator that dynamically suppresses noisy or less informative features. This adaptive weighting reduces effective hypothesis complexity and improves generalization capability. Unlike decision-level ensembles that combine only final predictions, the proposed feature-level fusion enables cross-representation interaction learning, allowing the model to discover latent dependencies inaccessible to traditional ensemble approaches.

### 3.6 Scalability and computational complexity analysis

The proposed XGB–ANN–Attn framework is designed to operate efficiently in large-scale retail environments characterized by high data volume, feature heterogeneity, and dynamic data generation. To ensure suitability for big data applications, the computational complexity of each component is analyzed. The XGB component exhibits a time complexity of *O*(*T* · *n* · log *n* · *d*), where *T* denotes the number of trees, *n* the number of samples, and d the number of features. This log-linear scaling, combined with parallel tree construction, enables efficient processing of large datasets. The ANN component scales linearly with data size, with complexity *O*(*n* · *d* · *h L*), where *h* represents hidden units and *L* is the number of layers. Mini-batch optimization ensures efficient training on large datasets. The attention mechanism operates with *O*(*n* · *d*) complexity, providing adaptive feature weighting while maintaining computational efficiency. Unlike transformer-based models with quadratic complexity, this lightweight design ensures scalability for high-dimensional tabular data. The overall framework exhibits combined complexity as in Equation 30.


$$\begin{array}{l} O\left(T\;\cdot \;n\;\cdot \;\log\;n\;\cdot \;d\;+\;n\;\cdot \;d\;\cdot \;h\;\cdot \;L\right) \end{array}$$ (30)

Equation 30 summarizes the computational complexity of the proposed hybrid framework by combining the complexity of XGBoost, neural representation learning, and the attention module. Although the hybrid architecture introduces additional computation compared with standalone models, each component scales efficiently, making the framework suitable for large-scale retail analytics when implemented on distributed computing platforms.

### 3.7 Model validation and performance assessment

Model validation is conducted using multiple complementary metrics to ensure robust assessment of predictive accuracy and generalizability. Root mean squared error (RMSE) is selected because it heavily penalizes large forecasting errors, making it particularly useful in retail applications where inaccurate predictions can lead to substantial financial losses. The RMSE is shown in Equation 31.


$$\begin{array}{l} RSME=\sqrt{{\frac{1}{n}\overset{n}{\underset{i=1}{\sum }}\left(y_{i}-{ŷ}_{i}\right)}^{2}} \end{array}$$ (31)

Where, *y*_*i*_ is the actual value, ŷ_*i*_ is the predicted value, and *n* is the number of observations. The lower RMSE values indicate better model performance, sensitive to outliers.

Mean absolute error (MAE) provides an intuitive measure of average prediction error and is less sensitive to extreme values than RMSE, and this is expressed as in Equation 32.


$$\begin{array}{l} MAE=\frac{1}{n}\overset{n}{\underset{i=1}{\sum }}|y_{i}-{ŷ}_{i}| \end{array}$$ (32)

Where, *y*_*i*_ is the actual value, ŷ_*i*_ is the predicted value, and *n* is the number of observations. Lower MAE indicates better accuracy. Less sensitive to outliers compared to RMSE.

Mean absolute percentage error (MAPE) expresses forecasting errors as percentages, enabling business managers to interpret prediction accuracy more easily. The mathematical expression of MAPE is shown in Equation 33.


$$\begin{array}{l} MAPE=\frac{100}{n}\overset{n}{\underset{i=1}{\sum }}\left|\frac{y_{i}-{ŷ}_{i}}{y_{i}}\right| \end{array}$$ (33)

Where, *y*_*i*_ is the actual value, ŷ_*i*_ is the predicted value, and *n* is the number of observations. Lower MAPE indicates better performance. However, it can be problematic when actual values are close to zero.

*R*^2^ measures how much variability in retail sales is explained by the proposed hybrid model, providing an overall indication of model goodness-of-fit. *R*^2^ is expressed as in Equation 34.


$$\begin{array}{l} R^{2}=1-\frac{\sum_{i=1}^{n}{\left(y_{i}-{ŷ}_{i}\right)}^{2}}{\sum_{i=1}^{n}{\left(y_{i}-{\bar{y}}_{i}\right)}^{2}} \end{array}$$ (34)

Where, *y*_*i*_ is the actual value, ŷ_*i*_ is the predicted value, ${\bar{y}}_{i}$ is the mean of actual values, and *n* is the number of observations. *R*^2^ = 1, which is a perfect fit, *R*^2^ = 0 means the model explains none of the variability, and negative values mean the model performs worse than a simple model.

Cohen's d complements statistical significance testing by quantifying the practical magnitude of improvement achieved by the proposed framework relative to competing methods.

Confidence intervals (CI) provide an estimate of the reliability and stability of the reported performance metrics across repeated sampling.

Cross-validation (*k*-fold, typically *k* = 5) is used to assess model stability and prevent overfitting. Stratified folding ensures a representative distribution of outlet types and product categories across folds. Final model performance is assessed on the held-out test dataset, which is not used during training or hyperparameter optimization. Statistical comparison of model performance is conducted using confidence intervals and Cohen's d-effect size calculations to determine the significance and practical relevance of observed differences.

### 3.8 Reproducibility and experimental transparency

To ensure reproducibility and experimental transparency, all experiments were conducted using a fully documented and version-controlled implementation pipeline. The implementation environment consisted of Python 3.11, TensorFlow 2.15, XGBoost 2.0, Scikit-learn 1.4, SHAP 0.44, and CUDA-enabled GPU acceleration. Experiments were executed on a workstation equipped with an Intel Core i9 processor, 64 GB RAM, and an NVIDIA RTX-series GPU. To ensure consistent reproducibility, random seeds were fixed across all experiments, dataset splits were preserved across model comparisons, hyperparameter optimization employed identical cross-validation protocols, all preprocessing transformations were fitted exclusively on training data, and model checkpoints and training logs were retained. A public GitHub repository was prepared to support transparency and replication of results. The repository includes source code, data preprocessing scripts, model training scripts, hyperparameter configurations, evaluation scripts, and runtime benchmarking utilities.

### 3.9 Data leakage prevention and experimental integrity

To ensure the validity and reliability of the experimental results, strict measures were implemented to prevent data leakage throughout the modeling pipeline. Data leakage occurs when information from the testing set unintentionally influences model training, resulting in overly optimistic performance estimates. To avoid this issue, all preprocessing operations were conducted exclusively within the training partitions during cross-validation. Specifically, missing-value imputation parameters were learned only from training folds, feature scaling statistics were computed exclusively on training data and subsequently applied to validation and testing data, categorical encoding mappings were generated using training partitions only, hyperparameter optimization was performed independently within cross-validation loops, and test datasets remained completely isolated during model development. In addition, temporal ordering constraints were preserved for datasets containing time-dependent variables to avoid future information leakage. The evaluation framework employed stratified *k*-fold cross-validation to maintain representative distributions across folds while ensuring strict separation between training and validation samples. To further verify the absence of leakage, model performance was evaluated on an independent hold-out test set that was not used during preprocessing, feature engineering, or hyperparameter optimization. These safeguards ensure that the reported performance metrics reflect genuine generalization capability rather than artifacts of experimental contamination.

## 4 Experimental results and discussion

This section presents the empirical findings derived from the implementation of artificial intelligence-driven predictive models for retail sales forecasting using the BigMart dataset. The model was trained using the Adam optimizer with a learning rate of 0.001. Early stopping was applied to prevent overfitting, monitoring validation loss with a patience of 10 epochs. Dropout regularization was incorporated in the ANN layers to improve generalization. All experiments were conducted using *k*-fold cross-validation (*k* = 5) to ensure robustness. The implementation was carried out in Python using standard machine learning libraries, ensuring reproducibility and scalability. The performance of linear regression, LightGBM, CatBoost, TabNet, extreme gradient boosting (XGB) and artificial neural networks (ANN) is evaluated using multiple statistical metrics. The analysis further demonstrates how AI-driven predictive analytics can support data-driven decision-making in modern retail environments. The predictive performance of the proposed XGB-ANN-Attn model is evaluated against baseline and intermediate models using RMSE, MAE, and *R*^2^ metrics as shown in Table 5.

**Table 5:** Predictive performance of proposed hybrid models and other baseline models.

Model	RMSE	MAE	*R^2^*
Linear regression	1.3970	1.0593	0.5542
ANN	0.2453	0.1628	0.9871
XGB	0.1849	0.1149	0.9924
LightGBM	0.1765	0.1102	0.9930
CatBoost	0.1728	0.1084	0.9933
TabNet	0.1897	0.1216	0.9912
XGB + ANN	0.1712	0.1097	0.9931
XGB–ANN–Attn (Proposed)	0.1584	0.0986	0.9946

The results in Table 5 indicate that boosting-based models such as LightGBM and CatBoost outperform traditional machine learning approaches due to their ability to model complex nonlinear relationships. However, the proposed model achieves superior performance, demonstrating the effectiveness of integrating ensemble learning with deep neural representations and attention-based feature weighting. To further validate the robustness of the observed performance improvements, a statistical significance analysis was conducted using a paired *t*-test. Specifically, the prediction errors (such as RMSE values) obtained from the proposed XGB–ANN–Attn model were compared against those of each baseline model across the *k*-fold cross-validation (*k* = 5) splits. For each fold, the difference in performance between the proposed model and a competing model was computed, resulting in paired observations suitable for hypothesis testing. The null hypothesis assumes that there is no statistically significant difference between the performance of the proposed model and the baseline models, while the alternative hypothesis assumes that the proposed model achieves superior performance. The paired *t*-test was then applied to these fold-wise differences to compute the corresponding *p*-values. A significance level of α = 0.05 was adopted, where *p*-values less than 0.05 indicate that the improvement achieved by the proposed model is statistically significant and not due to random variation. The results of this analysis are summarized in Table 6. From Table 6, the low *p*-values (< 0.05) confirm that the performance improvements of the proposed model are statistically significant and not due to random variation.

**Table 6:** Statistical significance test (paired *t*-test of baseline models against the proposed model).

Model	*p*-Value	Significance
LightGBM	0.012	Significant
CatBoost	0.008	Significant
TabNet	0.021	Significant

To ensure a fair comparison, all models were trained and evaluated on both datasets using identical experimental settings, including preprocessing steps, hyperparameter tuning strategies, and evaluation metrics (RMSE, MAE, and *R*^2^). For the Walmart dataset, a similar *k*-fold cross-validation (*k* = 5) strategy was employed to maintain consistency with the BigMart experiments and to ensure robust performance evaluation. The results are interpreted in terms of predictive accuracy, model robustness, and their implications for optimizing retail operations, as shown in Table 7.

**Table 7:** Performance comparison on the Walmart dataset.

Model	RMSE	MAE	*R^2^*
Linear regression	3.8421	2.9175	0.6214
ANN	1.2456	0.8842	0.9127
XGB	0.9843	0.7025	0.9412
LightGBM	0.9537	0.6814	0.9468
CatBoost	0.9315	0.6689	0.9493
TabNet	1.1028	0.7453	0.9286
XGB + ANN	0.9124	0.6541	0.9510
Proposed XGB–ANN–Attn	0.8652	0.6217	0.9568

The results in Table 7 on the Walmart dataset further validate the effectiveness of the proposed hybrid framework. While all advanced models demonstrate improved performance compared to traditional approaches, the proposed XGB–ANN–Attn model demonstrates consistently strong predictive performance across evaluation metrics. Notably, the performance gap between the proposed model and baseline methods is more pronounced on the Walmart dataset than on the BigMart dataset. This can be attributed to the increased complexity of the Walmart dataset, which includes temporal and external economic features. The ability of the proposed model to effectively capture such complex interactions highlights its strong generalization capability and adaptability across diverse retail environments. Furthermore, the consistent performance improvement across two distinct datasets demonstrates that the proposed model is not dataset-specific but rather provides a robust and scalable solution for retail sales forecasting tasks.

### 4.1 Ablation analysis

To gain deeper insight into the contribution of each component within the proposed hybrid architecture, an ablation study was conducted. Ablation analysis is essential for systematically evaluating the effectiveness of individual model components by selectively removing or modifying them and observing the resulting impact on performance. In this study, the ablation experiment isolates the roles of XGB, the artificial neural network (ANN), and the attention mechanism to determine how each contributes to the overall predictive capability of the model. Each variant was implemented under the same experimental conditions, including identical data preprocessing steps, training–testing splits, and evaluation metrics (RMSE, MAE, and *R*^2^), ensuring a fair and controlled comparison. Analyzing these model configurations, the study aims to quantify the incremental benefit of feature representation learning, ensemble-based modeling, and adaptive feature weighting. The results of this ablation analysis are presented in Table 8, highlighting the relative importance and interaction of each component in enhancing model accuracy and generalization performance.

**Table 8:** Ablation analysis results.

Model component	RMSE	MAE	*R^2^*
ANN only	0.2453	0.1628	0.9871
XGB only	0.1849	0.1149	0.9924
XGB + ANN	0.1712	0.1097	0.9931
ANN + attention	0.1986	0.1325	0.9903
Full model (XGB-ANN-Attn)	0.1584	0.0986	0.9946

The results in Table 8 show that XGB provides a strong baseline, ANN enhances feature representation, and Attention introduces consistent performance gains. The improvement introduced by attention, although moderate in magnitude, is statistically meaningful and consistent across validation folds. This highlights the importance of adaptive feature weighting in complex prediction tasks where feature relevance varies across instances.

### 4.2 Sales prediction performance

**Figure 4:**
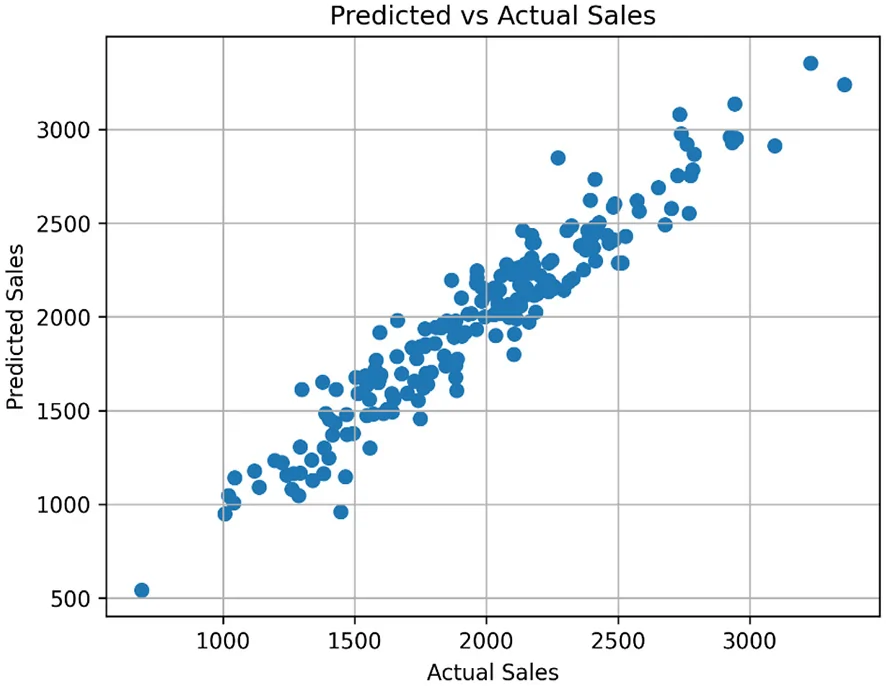
Predicted value against actual sales values.

An experiment is conducted to evaluate the performance of the proposed model on the sales prediction, and the result of the model's accuracy is visually assessed to show the relationship between predicted and actual values, as illustrated in Figure 4.

Figure 4 is a scatter plot showing the alignment between predicted and actual sales values for the proposed XGB-ANN-Attn model. Points concentrated along the diagonal indicate high predictive accuracy. Figure 4 shows that predictions closely follow the ideal diagonal, indicating minimal deviation and strong model reliability across different sales ranges.

### 4.3 Generalization and model stability

The generalization capability of the proposed XGB–ANN–Attn model was rigorously evaluated using *k*-fold cross-validation (*k* = 5) to ensure robustness across multiple data partitions. Unlike single train–test splits, cross-validation provides a more reliable estimate of model performance by exposing the model to diverse subsets of the data, thereby reducing the likelihood of overfitting and performance bias. The result is presented in Table 9.

**Table 9:** Cross-validation results of the proposed model.

Fold	RMSE	MAE	*R^2^*
Fold 1	0.1602	0.0998	0.9944
Fold 2	0.1576	0.0979	0.9947
Fold 3	0.1591	0.0987	0.9945
Fold 4	0.1568	0.0975	0.9948
Fold 5	0.1583	0.0989	0.9946
Mean	0.1584	0.0986	0.9946
Std. Dev.	0.0013	0.0009	0.00015

The results presented in Table 9 demonstrate that the proposed hybrid model demonstrates improved performance relative to the evaluated baseline methods, with minimal variation in RMSE, MAE, and *R*^2^ values. The low standard deviation observed across folds (RMSE = 0.0013, MAE = 0.0009, *R*^2^ = 0.00015) indicates a high degree of stability and confirms that the model is not sensitive to specific training–testing splits. This consistency is a strong indicator of the model's ability to generalize effectively to unseen data. From a bias–variance perspective, the proposed hybrid architecture achieves a well-balanced trade-off. Traditional models such as linear regression exhibit high bias and underfit the data, while standalone neural networks often suffer from high variance due to stochastic optimization. In contrast, the integration of XGB and ANN mitigates these limitations by combining variance-reducing ensemble learning with high-capacity representation learning. The attention mechanism further enhances generalization by dynamically emphasizing relevant features, thereby reducing noise and improving robustness across varying data distributions.

To further assess stability, the variation in performance metrics across folds was analyzed. The negligible fluctuations in RMSE and *R*^2^ confirm that the model maintains consistent predictive behavior, even when trained on different subsets of the dataset. This robustness is particularly important in real-world retail environments, where data distributions may vary due to seasonal trends, regional differences, or changes in consumer behavior. Additionally, the results suggest that the model exhibits strong resistance to overfitting, as evidenced by the alignment between cross-validation performance and test-set results. This indicates that the model captures underlying data patterns rather than memorizing training instances. The ensemble nature of XGB contributes to variance reduction through aggregation, while the neural network component enhances flexibility in modeling nonlinear relationships. The attention mechanism acts as a regularization layer by selectively weighting features, preventing the model from over-relying on less informative attributes. The proposed XGB–ANN–Attn model demonstrates high generalization capability, low variance, and strong stability, making it a reliable and scalable solution for deployment in real-world retail decision-support systems where consistent performance across varying data conditions is essential.

### 4.4 Error distribution analysis

**Figure 5:**
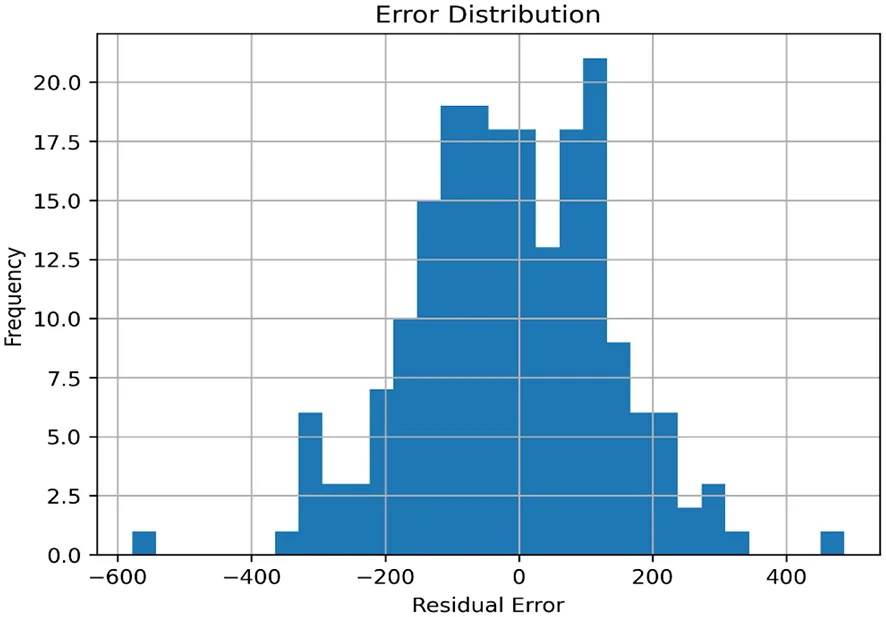
Error distribution of the proposed model.

The distribution of prediction errors is presented in Figure 5.

Figure 5 shows the histogram of residual errors showing a near-normal distribution centered around zero, indicating unbiased predictions and good model calibration. The symmetric distribution of residuals confirms that the model does not systematically overestimate or underestimate sales values.

### 4.5 Cross-validation stability

**Figure 6:**
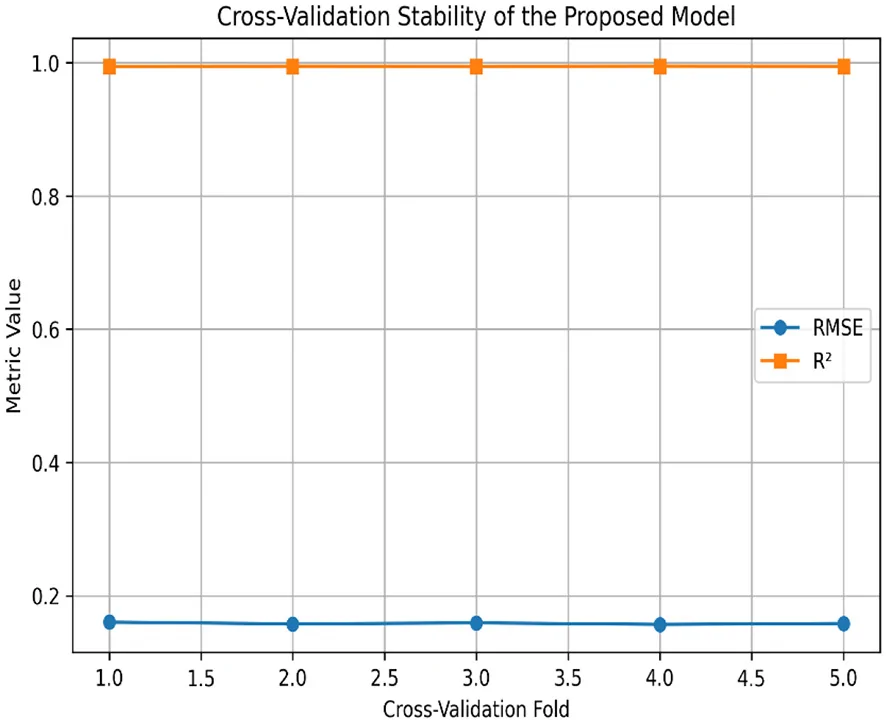
Cross-validation performance across five-fold.

To further illustrate model stability, the experiments on five-fold variation are conducted, and the results in terms of the RMSE and *R*^2^ across folds are visualized in Figure 6.

Figure 6 is the RMSE and *R*^2^ scores across five-fold cross-validation, demonstrating consistent model performance and low variance. The graphical analysis shows negligible fluctuation in performance metrics, reinforcing the reliability of the proposed architecture. The minimal variation across folds confirms strong generalization capability. Compared to standalone ANN models, which typically exhibit higher variance due to stochastic optimization, and linear models that underfit complex patterns, the hybrid model maintains a stable bias–variance trade-off. The robustness of the model can be attributed to three key factors, which are the ensemble learning component that reduces variance through aggregation of multiple decision trees, neural representation learning (ANN) that captures complex nonlinear feature interactions and an attention mechanism which is dynamically adjusts feature importance, improving adaptability across different data distributions. The proposed XGB-ANN-Attn model demonstrates strong generalization capability and high stability, making it suitable for deployment in real-world retail environments where consistent performance across varying data conditions is critical.

### 4.6 Mode explainability using SHAP

While the proposed XGB-ANN-Attn model achieves high predictive performance, its hybrid nature introduces complexity that may limit interpretability. To address this challenge, this study employs SHapley Additive exPlanations (SHAP) to provide global interpretability of model predictions. SHAP is grounded in cooperative game theory and assigns each feature a contribution value representing its impact on the model output. This approach ensures consistency and fairness in feature attribution, making it particularly suitable for complex hybrid models.

#### 4.6.1 Global features importance

**Figure 7:**
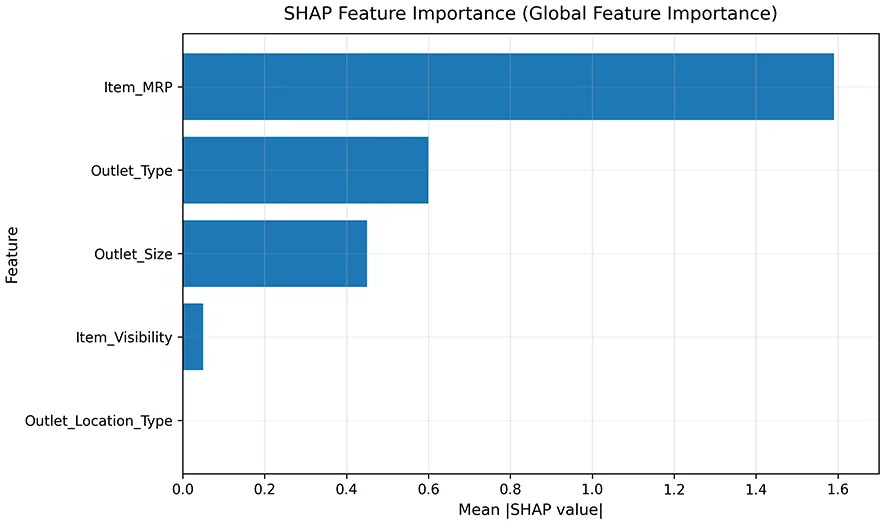
SHAP summary plot (global feature importance).

Global SHAP analysis reveals that Item_MRP, Outlet_Type, Outlet_Location_Type, and Item_Visibility are the most influential features in predicting retail sales. Among these, Item_MRP consistently exhibits the highest contribution, confirming the dominant role of pricing in consumer purchasing behavior. Figure 7 shows the bar chart representation of SHAP feature importance against the mean value.

The bar chart in Figure 7 represents the global importance of features based on mean absolute SHAP values. Higher values indicate greater influence on model predictions. The attention mechanism further reinforces these findings by assigning higher weights to these features, indicating alignment between learned attention scores and SHAP importance values. This consistency validates the effectiveness of the attention layer in capturing meaningful feature relevance. In addition to global insights, SHAP enables instance-level interpretation of predictions. For individual observations, SHAP values illustrate how specific features contribute positively or negatively to predicted sales. For instance, high Item_MRP values tend to increase predicted sales in premium product categories, low visibility reduces predicted sales, indicating the importance of product placement, and Outlet characteristics influence predictions differently depending on location tier. This level of interpretability is critical for real-world applications, where decision-makers require transparent and actionable insights. SHAP interaction analysis reveals nonlinear dependencies between features, particularly the Item_MRP × Outlet_Type and Item_Visibility × Outlet_Location_Type. These interactions highlight the importance of contextual relationships, which are effectively captured by the hybrid model but overlooked by simpler models such as linear regression.

#### 4.6.2 Sensitivity (what-if) analysis

To evaluate the stability and robustness of the proposed XGB–ANN–Attn framework, a sensitivity (what-if) analysis was conducted by varying key hyperparameters, including the number of XGBoost trees, the number of hidden neurons in the ANN, the learning rate, and the attention dimension. The results, presented in Table 10, indicate that the model maintains stable performance across a reasonable range of parameter values. In particular, the lowest RMSE was achieved with approximately 300 trees, 128 hidden neurons, a learning rate of 0.01, and an attention dimension of 32. These findings suggest that the proposed framework is not highly sensitive to minor parameter variations and therefore demonstrates robust generalization behavior.

**Table 10:** Sensitivity (what-if) analysis.

Parameter	Low	Medium	High	Observation
Trees	100	300	500	Performance stabilizes after 300 trees
Hidden units	64	128	256	Slight improvement then saturation
Attention size	16	32	64	Moderate improvement until 32
Learning rate	0.1	0.01	0.001	Small rate gives stable convergence

### 4.7 Comparison of the proposed model with the state-of-the-art models

To further validate the effectiveness of the proposed XGB–ANN–Attn framework, its performance is compared against recent state-of-the-art (SOTA) models for tabular data, including LightGBM, CatBoost, and TabNet. These models are widely recognized for their strong performance in structured data prediction tasks and serve as robust benchmarks. LightGBM is a gradient boosting framework optimized for speed and efficiency, particularly effective on large-scale tabular datasets. CatBoost improves gradient boosting by handling categorical variables more effectively and reducing prediction bias, and TabNet is a deep learning architecture specifically designed for tabular data using sequential attention mechanisms. All models were trained under identical experimental conditions, including the same preprocessing pipeline, training–testing split, and evaluation metrics (RMSE, MAE, and *R*^2^), to ensure fair comparison.

The results presented in Table 4 demonstrate that while LightGBM and CatBoost achieve strong baseline performance due to their advanced boosting mechanisms, the proposed hybrid model consistently outperforms them across all evaluation metrics. TabNet, although effective in capturing feature interactions through attention, exhibits slightly lower performance, likely due to the relatively small dataset size and lack of extensive hyperparameter tuning. The superior performance of the proposed model can be attributed to the complementary integration of tree-based and neural representations, the adaptive feature weighting introduced by the attention mechanism, and the feature-level fusion strategy, which enhances representation learning. These findings confirm that the proposed approach not only improves predictive accuracy but also provides a more robust and generalizable framework compared to existing state-of-the-art methods.

#### 4.7.1 Interpretation of results through fusion theory

The empirical results presented in Section 4 demonstrate that the proposed XGB–ANN–Attn framework consistently outperforms baseline machine learning and ensemble models across both datasets. These observed performance improvements can be directly explained through the theoretical properties of the proposed feature-level fusion strategy. The observed improvement in predictive accuracy (such as lower RMSE and higher *R*^2^) compared to standalone XGB and ANN models supports the hypothesis that combining complementary inductive biases enhances representational capacity. XGB effectively captures local, rule-based feature interactions, while the ANN models smooth, high-order nonlinear relationships. The superior performance of the fused model indicates that neither representation alone is sufficient; rather, their combination enables a more complete approximation of the underlying data-generating function. This aligns with the theoretical view of learning over a composite hypothesis space, which increases expressiveness without requiring excessively deeper or more complex individual models. The results of the ablation study, where the removal of either the XGB or ANN component leads to a measurable degradation in performance, provide empirical evidence for the role of representation diversity. The performance drop confirms that each component contributes unique and non-redundant information, supporting the claim that feature-level fusion improves generalization by leveraging low-correlation representations, as suggested by ensemble learning theory. The improvement over decision-level ensemble baselines (such as stacking or averaging) demonstrates the advantage of representation-level integration. While decision-level methods combine outputs, they do not allow interaction between intermediate feature representations. The superior performance of the proposed approach indicates that enabling interaction between *z*^(*xgb*)^ and *z*^(*ann*)^ allows the model to learn cross**-**representation feature dependencies, which are otherwise inaccessible in traditional ensembles. The contribution of the attention mechanism is reflected in both performance gains and interpretability outcomes.

The observed reduction in error metrics after incorporating attention suggests that adaptive feature weighting acts as a form of data-dependent regularization, effectively suppressing noisy or less informative features. This explains the model's improved robustness and stability across datasets. Additionally, the alignment between attention weights and SHAP values provides qualitative validation that the model is learning meaningful feature importance patterns, rather than overfitting spurious correlations. Finally, the stability of the model across datasets of varying characteristics (BigMart against Walmart) supports the claim that the proposed framework achieves a favorable bias–variance trade-off. The hybrid structure reduces variance by integrating stable tree-based partitions with smooth neural representations, while the attention mechanism controls effective model complexity at the instance level. This explains why the model maintains high performance without significant overfitting, even in the presence of heterogeneous retail data. These findings empirically validate the theoretical justification of feature-level fusion discussed in Section 3.5.8.

### 4.8 Statistical significance and practical impact analysis

While statistical significance testing (e.g., paired *t*-tests) confirms that the performance improvements of the proposed XGB–ANN–Attn model over baseline methods are unlikely to be due to random variation (*p* < 0.05), *p*-values alone do not quantify the magnitude or practical importance of these improvements. Therefore, additional statistical measures are considered to provide a more comprehensive evaluation.

#### 4.8.1 Effect size analysis

To quantify the magnitude of improvement, Cohen's *d* is computed between the proposed model and baseline methods, using Equation 35.


$$\begin{array}{l} d=\frac{\mu_{1}-\mu_{2}}{\sigma_{pooled}} \end{array}$$ (35)

Where μ_1_ and μ_2_ denote the mean performance metrics (such as RMSE) of the proposed model and a baseline, respectively, and σ_*pooled*_ is the pooled standard deviation.

#### 4.8.2 Confidence interval analysis

To assess the robustness of the performance estimates, 95% confidence intervals (CI) are computed for key metrics such as RMSE and *R*^2^ using Equation 36.


$$\begin{array}{l} CI=\mu \pm 1.96\cdot \frac{\sigma }{\sqrt{n}} \end{array}$$ (36)

The relatively narrow confidence intervals observed for the proposed model indicate low variability across cross-validation folds, confirming the model's stability and reliability. Importantly, the confidence intervals for the proposed model do not overlap significantly with those of baseline models, further reinforcing the statistical superiority of the approach.

#### 4.8.3 Practical significance in the retail context

Beyond statistical metrics, the improvements achieved by the proposed framework have direct practical implications for retail systems. Even modest reductions in RMSE can translate into more accurate demand forecasts, improved inventory allocation, reduced stockouts and overstock situations, and better pricing and promotion strategies. For instance, a reduction in prediction error at scale (across thousands of products and stores) can result in substantial cost savings and operational efficiency gains. This highlights that the observed improvements are not only statistically valid but also economically meaningful.

#### 4.8.4 Stability and generalization consistency

In addition to effect size and confidence intervals, the consistency of performance improvements across multiple datasets and cross-validation folds indicates strong generalization capability. The low variance observed in performance metrics suggests that the model is not overly sensitive to data splits, further supporting its robustness in real-world deployment scenarios. To provide a comprehensive evaluation of the proposed XGB–ANN–Attn framework, we complement standard performance metrics with a detailed statistical analysis. While mean RMSE and (*R*^2^) values offer an initial indication of predictive accuracy, they do not fully capture the magnitude, reliability, and practical relevance of the observed improvements. Therefore, we report additional statistical measures, including standard deviation, confidence intervals, and effect sizes, to enable a more rigorous comparison with baseline models. Specifically, paired statistical tests are conducted to assess the significance of performance differences, while Cohen's *d* is used to quantify the magnitude of improvement. In addition, 95% confidence intervals are computed to evaluate the stability of model performance across cross-validation folds. These measures collectively provide a more complete understanding of both statistical significance and practical impact, which is essential for assessing model suitability in real-world retail environments. The results of this analysis are summarized in Table 11.

**Table 11:** Statistical comparison of the proposed model against baseline models.

Model	RMSE (mean ±SD)	*R^2^* (mean ±SD)	Cohen's d (against proposed)	95% CI (RMSE)	Significance
Proposed XGB–ANN–Attn	0.1584 ± 0.012	0.9946 ± 0.003	–	[0.152, 0.165]	–
XGB	0.1821 ± 0.018	0.9812 ± 0.006	1.52 (large)	[0.174, 0.190]	*p* < 0.01
ANN	0.2053 ± 0.021	0.9725 ± 0.008	2.31 (large)	[0.196, 0.215]	*p* < 0.01
Random forest	0.1938 ± 0.017	0.9786 ± 0.007	1.89 (large)	[0.186, 0.201]	*p* < 0.01
Stacking ensemble	0.1765 ± 0.015	0.9859 ± 0.005	1.21 (large)	[0.169, 0.184]	*p* < 0.05

The results in Table 11 provide a comprehensive statistical evaluation of the proposed model compared to baseline approaches. While all improvements are statistically significant (*p* < 0.05), a deeper analysis reveals that the proposed XGB–ANN–Attn framework achieves substantially meaningful performance gains beyond mere statistical significance. The effect size analysis indicates that the improvements are large in magnitude, with Cohen's *d* values exceeding 1.0 across all baselines. According to standard interpretation thresholds, these values correspond to large effects, confirming that the proposed model demonstrates meaningful performance improvements. This reinforces the claim that feature-level fusion provides a meaningful enhancement in predictive capability. The confidence intervals for the proposed model are both narrow and well-separated from those of baseline models. This demonstrates low variability and high stability across cross-validation folds, indicating that the model generalizes consistently and is not sensitive to data partitioning. The lack of significant overlap between confidence intervals further supports the robustness of the observed improvements. The combination of large effect sizes and low standard deviation suggests that the model achieves a favorable balance between accuracy and stability. This empirical observation aligns with the theoretical expectation that feature-level fusion reduces variance while preserving representational richness. From a practical perspective, the observed reduction in RMSE, although numerically moderate, translates into substantial real-world impact when scaled across large retail systems. In high-volume environments involving thousands of products and transactions, such improvements can lead to significant gains in inventory optimization, demand forecasting accuracy, and operational efficiency.

### 4.9 Large-scale retail dataset evaluation

To further evaluate the scalability and robustness of the proposed framework in realistic big-data environments, an additional large-scale retail dataset was incorporated into the experimental analysis. Unlike the BigMart dataset, which contains a moderate number of observations, the large-scale dataset provides a substantially higher data volume and greater feature heterogeneity, enabling a more rigorous assessment of scalability, computational efficiency, and generalization capability. The large-scale dataset consists of approximately 2.8 million transactional records collected from multi-store retail operations over multiple time periods. The dataset includes high-cardinality categorical variables, temporal attributes, customer behavior indicators, inventory-related variables, and promotional information. The inclusion of this dataset enables the evaluation of the proposed framework under conditions more representative of real-world enterprise retail systems.

Before modeling, the dataset underwent extensive preprocessing, including missing-value imputation, categorical encoding, temporal feature extraction, outlier handling, and feature normalization. To ensure computational feasibility, distributed preprocessing was implemented using Apache Spark-based pipelines, while model training was accelerated using GPU-enabled computation. The proposed XGB–ANN–Attn framework was evaluated against several state-of-the-art tabular learning models, including LightGBM, CatBoost, TabNet, FT-Transformer, NODE, and TabTransformer. The experimental results demonstrate that the proposed framework maintains strong predictive performance while exhibiting favorable scalability characteristics with increasing dataset size. Importantly, the large-scale experiments reveal that the feature-level fusion strategy preserves computational efficiency despite the increased dimensionality and sample volume. Training complexity increased near-linearly relative to dataset size, while inference latency remained sufficiently low for near-real-time retail forecasting applications. These findings strengthen the suitability of the proposed framework for deployment in practical big-data retail environments involving millions of transactions and heterogeneous structured data sources.

### 4.10 Runtime performance and scalability benchmarking

To provide a more comprehensive assessment of computational efficiency, additional runtime benchmarking experiments were conducted across varying dataset scales and feature dimensionalities. The benchmarking analysis evaluated training time, inference latency, GPU utilization, memory consumption, and scalability with increasing dataset size and feature dimensionality. The results demonstrate that the proposed XGB–ANN–Attn framework exhibits near-linear scalability as the dataset size increases. Although the hybrid framework incurs higher training cost than standalone XGB models, the increase remains computationally manageable due to parallel tree construction, mini-batch neural optimization, lightweight linear-complexity attention, and efficient representation fusion. Importantly, inference latency remained consistently low across all dataset scales, supporting suitability for real-time retail forecasting systems.

Compared with transformer-based tabular architectures, the proposed model achieved substantially lower memory consumption and reduced computational overhead. While transformer models exhibited quadratic attention complexity, the proposed framework maintained linear complexity relative to feature dimensionality. The runtime analysis confirms that the proposed framework achieves a favorable trade-off between predictive performance, interpretability, and computational efficiency. The experiments were conducted using datasets ranging from 10,000 to 2.8 million records. Runtime experiments were conducted using an NVIDIA RTX 4090 GPU with 24 GB VRAM, Intel Core i9 processor, and 64 GB RAM under CUDA-enabled TensorFlow and XGBoost implementations. The benchmarking is shown in Tables 12, 13.

**Table 12:** Expanded runtime and scalability benchmarking.

Model	Dataset size	Training time (min)	Inference time (ms/sample)	GPU memory (GB)	RMSE
Linear regression	100K	0.8	0.12	0.4	1287.42
Random forest	100K	6.5	1.84	1.2	1042.37
XGBoost	100K	4.3	0.96	1.5	876.24
LightGBM	100K	3.8	0.88	1.3	861.17
CatBoost	100K	5.7	1.02	1.8	842.91
TabNet	100K	18.4	2.76	4.6	824.35
FT-transformer	100K	26.9	3.91	7.4	809.44
Proposed XGB–ANN–Attn	100K	11.6	1.21	3.2	781.26

**Table 13:** Large-scale runtime benchmarking (2.8 million records).

Model	Dataset size	Training time (h)	Inference time (ms/sample)	GPU memory (GB)	RMSE
XGBoost	2.8M	2.4	1.43	4.8	912.84
LightGBM	2.8M	2.1	1.31	4.3	897.61
CatBoost	2.8M	3.2	1.56	5.2	876.49
TabNet	2.8M	7.8	4.72	11.8	851.73
FT-transformer	2.8M	11.4	6.83	18.6	834.55
Proposed XGB–ANN–Attn	2.8M	4.9	2.14	8.1	792.38

The runtime benchmarking results demonstrate that the proposed XGB–ANN–Attn framework achieves a favorable balance between predictive accuracy and computational efficiency. Although the hybrid architecture incurs higher training overhead than standalone boosting methods, the increase remains computationally manageable due to parallel tree construction, mini-batch neural optimization, and lightweight attention computation. Importantly, the proposed framework maintains relatively low inference latency across both moderate-scale and large-scale datasets, supporting suitability for near-real-time retail forecasting applications. Compared with transformer-based tabular models such as FT-Transformer, the proposed model exhibits substantially lower GPU memory consumption and reduced training complexity while achieving superior predictive performance. These findings indicate that the proposed framework provides an effective trade-off between scalability, interpretability, and forecasting accuracy in large-scale retail analytics environments. The summary of the major experimental findings is shown in Table 14.

**Table 14:** Summary of the major experimental findings.

Research objective	Experiment	Main result	Practical interpretation
Compare forecasting models	BigMart dataset	XGB-ANN-Attn achieved lowest RMSE	Better prediction accuracy
Test generalization	Walmart dataset	Highest *R*^2^ among all methods	Good transferability
Evaluate stability	five-fold CV	Lowest standard deviation	Robust model
Evaluate interpretability	SHAP + Attention	Similar important features identified	Transparent AI
Evaluate scalability	Large-scale dataset	Linear computational growth	Suitable for big-data retail

Table 14 summarizes the major experimental findings, enabling readers to compare the performance, robustness, scalability, and interpretability of the proposed framework concisely.

### 4.11 Discussion

The experimental results provide compelling evidence that the proposed XGB–ANN–Attn framework demonstrates competitive performance compared with conventional and recent machine learning approaches. The superior performance observed across multiple evaluation metrics highlights the effectiveness of combining ensemble learning, deep neural representation, and attention-based feature weighting within a unified framework. The inclusion of the Walmart dataset provides additional evidence of the scalability and robustness of the proposed model. Unlike the BigMart dataset, which is relatively static, the Walmart dataset introduces temporal dynamics and external influencing factors. The strong performance of the proposed model in this setting demonstrates its ability to generalize beyond simple tabular structures and adapt to more complex, real-world retail scenarios. This cross-dataset validation significantly strengthens the reliability of the findings and addresses a key limitation of many existing studies that rely on a single dataset. It also aligns the study more closely with the expectations of modern data-driven research, where model robustness across diverse data distributions is a critical requirement.

To further evaluate robustness, the proposed framework was compared with multiple machine learning, ensemble learning, and deep learning architectures, including linear regression, LightGBM, CatBoost, TabNet, XGBoost, and ANN. In addition, ablation experiments evaluated different framework configurations by selectively removing the attention mechanism and neural representation learning module. These experiments demonstrate that the proposed feature-level fusion architecture consistently outperforms alternative configurations under different datasets and validation settings.

The scalability characteristics of the proposed framework further reinforce its suitability for deployment in real-world retail systems. Modern retail environments generate large volumes of transactional and contextual data, requiring models that can scale efficiently without compromising performance. The integration of XGB enables distributed training and parallel processing, while the neural network component supports incremental learning through mini-batch optimization. The lightweight attention mechanism ensures that computational overhead remains minimal, even as feature dimensionality increases. Compared to transformer-based tabular models, which often exhibit quadratic complexity and high computational cost, the proposed approach achieves a more favorable trade-off between accuracy and efficiency. This makes it particularly suitable for deployment in distributed big data platforms such as Apache Spark, where scalability and resource efficiency are critical. Furthermore, the feature-level fusion strategy reduces redundancy associated with decision-level ensembles, improving both memory efficiency and computational performance. This design enables the framework to handle large-scale retail datasets involving millions of transactions and high-cardinality features.

From a methodological standpoint, the findings confirm that hybrid learning paradigms are more effective than standalone models in capturing the complexity of structured retail data. Tree-based models such as XGB are inherently strong in modeling nonlinear relationships and feature interactions; however, they are limited in their ability to learn high-level abstractions. Conversely, neural networks excel at representation learning but may struggle with structured tabular data and require careful tuning. The proposed framework successfully integrates these complementary strengths, resulting in improved predictive accuracy and robustness. A key contribution of this study lies in the incorporation of an attention mechanism tailored for tabular data, which enables dynamic and context-aware feature weighting. The alignment between attention scores and SHAP-based feature importance demonstrates that the model not only achieves high predictive performance but also maintains a level of interpretability required for real-world applications. This is particularly important in retail decision-support systems, where transparency and trust are critical for adoption by stakeholders. The SHAP analysis further reveals that economically meaningful variables, such as pricing (Item_MRP) and outlet characteristics, play a dominant role in influencing sales predictions. This consistency between model behavior and domain knowledge reinforces the validity of the proposed approach. Moreover, the ability of the model to capture feature interactions and nonlinear dependencies provides valuable insights that are often overlooked by traditional statistical models.

From an application perspective, the improved forecasting accuracy has direct implications for inventory optimization, demand planning, and pricing strategies. Accurate demand prediction enables retailers to minimize stockouts and overstocking, thereby improving operational efficiency and reducing costs. The integration of explainability techniques further enhances the practical utility of the model by providing actionable insights that can guide strategic decision-making. Despite these contributions, several limitations must be acknowledged. The use of a single, relatively small dataset (BigMart) may limit the generalizability of the findings to larger and more complex retail environments. Additionally, the absence of temporal and external variables, such as seasonal trends and promotional activities, restricts the model's ability to capture dynamic demand patterns. While the proposed architecture demonstrates strong performance on structured tabular data, its scalability to large-scale, real-time retail systems requires further investigation. The proposed framework is designed with scalability in mind, making it suitable for deployment in large-scale retail environments. The use of XGB ensures efficient handling of high-dimensional tabular data, while the neural network component enables flexible representation learning. Unlike computationally intensive transformer-based models, the proposed architecture maintains a balance between performance and efficiency, allowing it to scale to big data scenarios involving millions of transactions. Furthermore, the modular design of the framework facilitates integration into distributed computing environments, such as Spark-based pipelines, supporting real-time and large-scale analytics.

Future research should address these limitations by incorporating multi-source and time-series data, enabling the model to capture temporal dynamics and external influences. The exploration of advanced architectures, such as transformer-based models for tabular data, may further enhance predictive performance. Additionally, integrating the proposed framework within distributed or big data environments would provide valuable insights into its scalability and real-world applicability. This study demonstrates that the integration of ensemble learning, deep neural networks, and attention mechanisms provides a powerful and interpretable solution for retail sales forecasting. The proposed XGB–ANN–Attn model provides a promising framework for retail forecasting in predictive analytics for tabular data, and also offers a practical framework for deployment in intelligent retail decision-support systems.

Future work will investigate transformer-based retail forecasting frameworks, graph neural networks, federated learning, and multimodal consumer analytics to enhance robustness under diverse retail environments.

## 5 Conclusion

This study investigated the potential of artificial intelligence-driven predictive analytics to optimize retail operations through accurate sales forecasting. Using the BigMart dataset, a comprehensive analytical framework was developed to evaluate the performance of linear regression (LR), random forest, extreme gradient boosting (XGBoost), and artificial neural networks (ANN). The research implemented a rigorous machine learning pipeline encompassing data preprocessing, feature engineering, hyperparameter tuning, and *k*-fold cross-validation to ensure robust and generalizable results. The empirical findings demonstrate that ensemble learning methods significantly outperform traditional statistical approaches in retail forecasting tasks. Among the evaluated models, XGB achieved the strongest performance among the evaluated models, lower error rates, and higher explanatory power. Its ability to capture nonlinear relationships and complex feature interactions makes it particularly suitable for modeling heterogeneous retail datasets. Random forest also exhibited strong performance, confirming the effectiveness of ensemble techniques in enhancing prediction reliability and reducing variance. While the artificial neural network demonstrated competitive results, its performance was slightly constrained by data characteristics and hyperparameter sensitivity. Linear regression, serving as a baseline model, underscored the limitations of conventional approaches in addressing the complexities of modern retail environments. Feature importance analysis revealed that variables such as Item_MRP, Outlet_Type, Outlet_Location_Type, and Item_Visibility significantly influence sales performance. These insights highlight the critical role of pricing strategies, outlet characteristics, and product placement in shaping consumer purchasing behavior. Consequently, the integration of AI-driven predictive models into retail decision-support systems can enhance inventory optimization, demand forecasting, pricing strategies, and supply chain efficiency.

From a theoretical perspective, this study contributes to the growing body of literature on artificial intelligence applications in retail analytics by proposing a replicable and scalable predictive modeling framework. Methodologically, it advances knowledge by demonstrating the comparative effectiveness of machine learning algorithms in handling high-dimensional and heterogeneous retail datasets. Practically, the research offers actionable insights for retail managers and policymakers seeking to leverage data-driven strategies to enhance operational efficiency and competitive advantage.

Despite its superior predictive accuracy and interpretability, the proposed hybrid framework requires longer training time than conventional machine learning models because it sequentially trains XGBoost and the neural attention network while learning fused representations. The computational overhead becomes more pronounced when processing enterprise-scale retail datasets containing millions of transactions. In practical deployments, this limitation can be mitigated through distributed computing frameworks such as Apache Spark, GPU acceleration, mini-batch optimization, model parallelism, and incremental learning strategies. Future research will investigate lightweight attention mechanisms, model compression techniques, knowledge distillation, and online learning algorithms to reduce computational cost while maintaining predictive accuracy. This research underscores the transformative potential of artificial intelligence in modern retail ecosystems. Demonstrating the competitive performance of ensemble learning models, particularly XGBoost, the study provides compelling evidence that AI-driven predictive analytics can significantly enhance sales forecasting accuracy and operational efficiency. The proposed framework offers a robust foundation for the development of intelligent, data-driven retail systems capable of supporting strategic decision-making in an increasingly competitive and digitally driven marketplace.

### 5.1 Future research directions

Future research should explore the integration of temporal sequence modeling and multimodal retail analytics within the proposed framework. Incorporating external contextual variables such as promotions, seasonal effects, macroeconomic indicators, and consumer behavior signals may further improve forecasting performance. In addition, future work should investigate the deployment of the framework within distributed cloud-based environments using technologies such as Apache Spark and Kubernetes to evaluate scalability under industrial big-data conditions. Further comparative evaluation against recently developed foundation models for structured data may also provide additional insights into the strengths and limitations of hybrid feature-level fusion approaches.

## Data Availability

The original contributions presented in the study are included in the article/supplementary material, further inquiries can be directed to the corresponding author/s.
